# A Meta-Analysis on the Seroprevalence of Parvovirus B19 among Patients with Sickle Cell Disease

**DOI:** 10.1155/2019/2757450

**Published:** 2019-12-09

**Authors:** Sagad Omer Obeid Mohamed, Esraa Mohamed Osman Mohamed, Afnan Abugundul Ahmed Osman, Fatima Abdelhakam Abdellatif MohamedElmugadam, Gehad Abdelmonem Abdalla Ibrahim

**Affiliations:** Faculty of Medicine, University of Khartoum, Khartoum, Sudan

## Abstract

**Background:**

Parvovirus B19 (B19 V) infection had been reported to be more frequent with serious clinical outcomes in patients with sickle cell disease (SCD) than in the general population. There is a wide variation in data among the existing literature regarding the seroprevalence of B19 V in patients with SCD. These data require further summary and analyses for better accuracy. This systematic review and meta-analysis was done to estimate the seroprevalence of B19 V in patients with SCD.

**Methods:**

This study was conducted according to the Preferred Reporting Items for Systematic Reviews and Meta-Analyses (PRISMA) guidelines. The databases of MEDLINE/PubMed, Virtual Health Library (VHL), ScienceDirect, Google Scholar, and OpenGrey were used for the systematic search. The random-effects model was used to estimate the pooled prevalence with the corresponding 95% confidence interval (CI) using OpenMeta Analyst software. Publication bias was estimated based on Begg's test, Egger's test, and examination of the funnel plot. Subgroup analyses and metaregression were used to explore the moderators of heterogeneity between studies.

**Results:**

A total of 18 studies including 2890 patients were analyzed. The overall IgG seroprevalence of B19 V infection among patients with SCD was found to be 48.8% (95% CI 39.5%–58.0%). Evidence of publication bias was not detected. Evidence of acute viral infection detected by positive IgM antibodies among the screened SCD patients was found in 8.30% (95% CI 5.20%–11.4%) of them. There was a statistically signiﬁcant association between seroprevalence of B19 V and geographical areas.

**Conclusion:**

There was a high prevalence of B19 V in patients with SCD. Healthcare providers need to be aware of the magnitude of B19 V infection in patients with SCD to ensure effective management. This review could provide a comprehensive view of B19 V prevalence in this susceptible population.

## 1. Introduction

Sickle cell disease (SCD) is the most common genetic hematological disorder characterized by the presence of a hemoglobin tetramer composed of mutated beta S-globin chains [1–4]. SCD is characterized by chronic hemolytic anemia and vaso-occlusion leading to infarction of multiple organs and tissues [4, 5]. Morbidity is considerably increased in SCD, and several disorders are more frequent in patients with SCD such as microbial infections, hyposplenism, dactylitis, acute chest syndrome (ACS), cholelithiasis, and neurological complications with transient ischemic attacks and stroke [1, 5, 6].

Parvovirus B19 (B19 V) is a small nonenveloped virus of a single-stranded DNA. It is a very stable virus and resistant to standard procedures for physical inactivation with detergents or heat [7–10]. B19 V infection had been reported globally, and it is most commonly transmitted as droplet infections through the respiratory secretion, or vertically through the placenta to the fetus, and through bone marrow and organ transplantations [10–12]. Diagnostic tests used for confirmation of B19 V include serum specific IgG antibodies testing which is used to confirm an exposure to B19 V infection, serum IgM antibodies testing which is recommended to diagnose acute viral infection and remain detectable several months after infection, and other diagnostic tests such as viral DNA detection by using the PCR technique [7, 11, 13].

B19 V infection had been reported to be more frequent with serious clinical outcomes in SCD patients than in the general population [5, 8, 11, 12, 14]. In SCD patients, an acute B19 V infection can precipitate prolonged vaso-occlusive crisis resulting in splenic sequestration, glomerulonephritis, cerebrovascular accident, myocarditis, and fatal bone marrow embolism [15].

Also, B19 V causes the well-known acute clinical event called transient aplastic crisis (TAC), where temporary erythrocyte aplasia with severe anemia occurs [1, 5, 8]. Although the outcome of TAC is mostly nonthreatening, some patients become extremely ill at presentation and should be treated by red cell transfusions to minimize the threat of circulatory collapse and heart failure due to severe anemia [16]. Once the immune response clears the infection, the red cell production resumes followed by lifelong immunity [5, 8, 17].

Many studies investigated the prevalence of this pathogenic virus in patients with SCD. However, these studies remain inconsistent with wide variation in the data obtained from these studies, and to the best of our knowledge, there is no meta-analysis of existing contemporary evidence on the seroprevalence of B19 V in patients with SCD. These data require further summary and analyses for better accuracy. The results could provide a comprehensive view of B19 V prevalence in this susceptible population and may contribute to its control and management.

## 2. Materials and Methods

This study was registered in the PROSPERO database with the protocol number (CRD42018115360). We conducted this systematic review and meta-analysis in accordance with the PRISMA statement guidelines (Preferred Reporting Items for Systematic Reviews and Meta-Analyses) [18]. In April 2019, a literature search using the bibliographic databases of MEDLINE/PubMed, Virtual Health Library (VHL), ScienceDirect, Google Scholar, and OpenGrey was conducted to identify and recruit all of the relevant studies without restriction regarding the publication period. The search strategy was formulated by using the words “Parvovirus” and “sickle cell” to ensure maximum coverage of possible literature. Furthermore, a manual search for other additional studies was performed using references cited in original selected study articles.

We included studies published in the English language, with sufficient information to estimate the seroprevalence rate of B19 V in patients with SCD. Study exclusion criteria included the following: case reports, case series, editorial letters, reviews, conference abstracts, studies lacking the data of interest, and studies conﬁned to specific subgroups of SCD patients (with complications, e.g., arthropathies or cerebral infarction). Four reviewers did screening of the titles and abstracts of the identified studies, assessed the full text of potentially eligible studies, and extracted the relevant data. Any disparity was resolved by discussion and consensus. Quality of the studies was assessed using a quality assessment tool for prevalence studies suggested by Hoy et al., which is a tool that addresses internal and external validity issues of prevalence studies based on combined criteria [19]. We extracted the following information using purpose-designed data extraction form each article: first author's name, year of publication, geographical location of the study, sample size, age groups of the patients, and reported prevalence B19 V infection detected by serological tests among the SCD patients.

### 2.1. Statistical Analysis

Extracted data were exported into OpenMeta Analyst version 10.10 software for analysis [20]. A meta-analysis of pooled prevalence with 95% CIs was carried out using a random-effects model due to high heterogeneity, and the results were displayed in a forest plot. When the number of pooled studies was small (up to 20), we used the Hartung–Knapp–Sidik–Jonkman (HKSJ) estimator to compute the random-effects analysis [21]. It has been reported that the HKSJ method outperforms the standard DerSimonian–Laird method when there is heterogeneity, and the number of the analyzed studies is small [21]. Publication bias was estimated by using StatsDirect software version 3.1.22 based on Begg's test, Egger's test, and visual examination of the funnel plot [22, 23]. We used subgroup analyses by the study region and metaregression for examining the effect of sample size and publication year to explore the reasons for heterogeneity between studies. The chi-square test was used to assess the differences between the categorical subgroups, and the significance level was set at 0.05.

## 3. Results

### 3.1. Study Identification and Characteristics

A total of 550 potentially relevant studies were retrieved during our database search. After screening the titles and abstracts of these articles, we excluded 500 articles that were obviously irrelevant or duplicated in the databases. The remaining 50 studies were retrieved for a full-text assessment. Full texts of these 50 studies were screened, and 32 studies of which were subsequently omitted because of low quality or lack of data to estimate the outcomes of interest. These excluded studies were 14 review articles [1, 3, 5, 7, 8, 11–13, 18, 24–28], ten studies had small number of patients with SCD [29–38], a study included patients with several types of chronic hemolytic anemia [39], four studies done among patients with TAC [40–43], a study analyzed all the acute admissions of patients with SCD to a district general hospital [44], a study included all patients attending some hospitals [45], and a study done among healthy blood donors [46].

**Figure 1:**
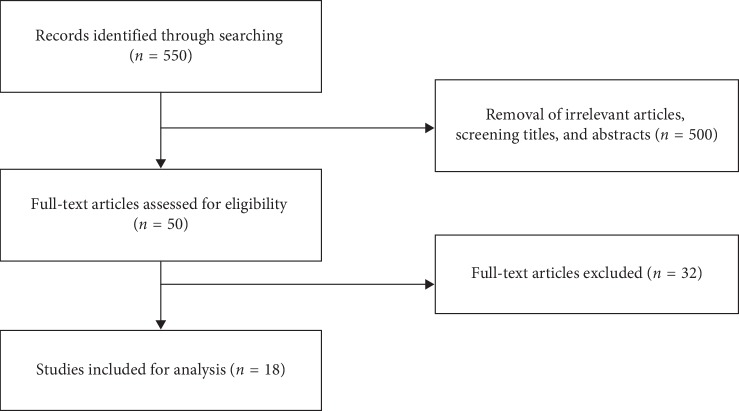
The flow diagram for the process of study selection and systematic review of literature.

Lastly, a total of 18 studies published from 1993 to 2016 representing 2890 patients which met the eligibility criteria were used for qualitative and quantitative syntheses: 10 studies from Africa [2, 6, 9, 10, 14, 47–51], 6 studies from the Americas [15, 17, 52–55], and 2 studies from Asia [56, 57] (Additional file 1. [Supplementary-material supplementary-material-1]). The kappa values for the selection of literature and data extraction were 0.7 and 0.8, respectively. The schematic flow of study identification and selection process is presented in Figure 1.

### 3.2. Epidemiology of B19 V Infection among Patients with SCD

**Figure 2:**
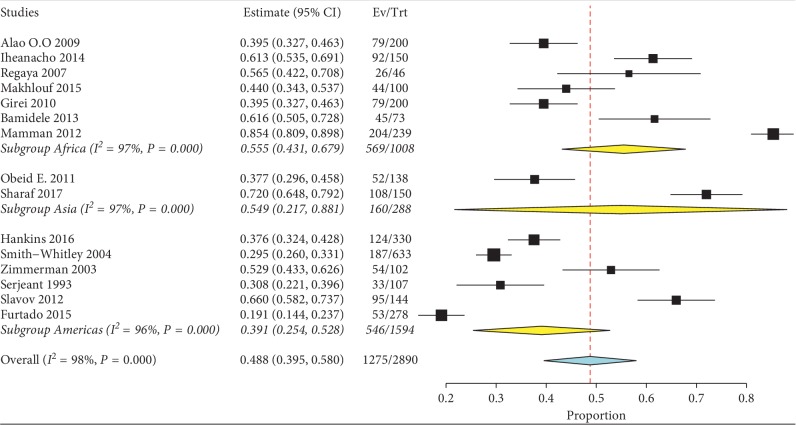
Pooled prevalence of B19 V infection among patients with sickle cell disease.

**Figure 3:**
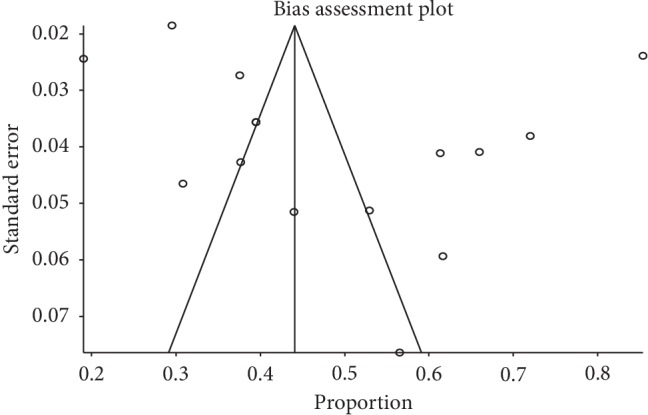
Funnel plot for publication bias assessment.

Meta-analysis for the included studies showed that the overall IgG seroprevalence from the random-effects model was 48.8% (95% CI 39.5%–58.0%) (Figure 2). The *I*-square test showed a high level of heterogeneity among the studies (*I*^2^ = 98%, *P* < 0.001), and the *t*-statistic for the degree of freedom (*N*−1 df = 14) was 2.144. No evidence of publication bias was detected on visual examination of the funnel plot (Figure 3) and from the results of Begg's test (*P*=0.46) and Egger's test (*P*=0.38). Evidence of acute viral infection detected by positive IgM antibodies was found only in 8.30% (95% CI 5.20%–11.4%) among the screened SCD patients.

### 3.3. Moderators of Heterogeneity

In subgroup analysis based on different study regions, the pooled prevalence of B19 V infection among patients was the highest in Africa, where it was 55.5% (95% CI 38.7%–77.2%). The pooled prevalence was 39.0% (95% CI 27.1%–50.9%) in the Americas and 54.9% (95% CI 21.3%–88.5%) in Asia (Figure 2). There was a significant difference in prevalence between different geographical areas (*X*^2^ = 140.4, *P* < 0.001) (Table 1).

In subgroup analysis based on different age groups, the pooled prevalence of B19 V infection was the highest in older age groups. The pooled prevalence was 42.0% (95% CI 29.6%–54.4%) among children with SCD, 72.0% (95% CI 64.8%–79.2%) among adults with SCD, and 56.5% (95% CI 46.5%–66.6%) among patients from the both age groups (Table 1). There was a significant difference in prevalence between different age groups (*X*^2^ = 102.1, *P* < 0.001) (Table 1).

**Figure 4:**
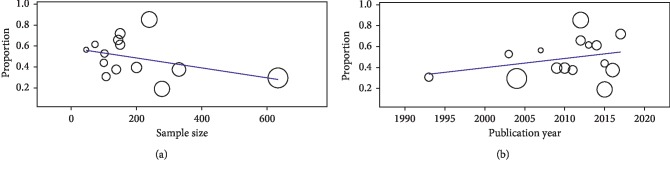
Metaregression scatter plots showing the correlation between seroprevalence, sample size, and publication year: (a) regression of sample size on prevalence; (b) regression of publication year on prevalence.

Furthermore, metaregression analyses were done to analyze whether the continuous variables (sample size and publication year) affected the heterogeneity in this meta-analysis. The results showed that sample size (*P*=0.118) and publication year (*P*=0.282) had no moderating effects on the outcome of this analysis and were not correlated with the effect size (Figure 4).

## 4. Discussion

During the disease course, patients with SCD are highly susceptible to infection with several microorganisms, including B19 V [5]. To the best of our knowledge, this study is the first meta-analysis of epidemiological studies on the seroprevalence of B19 V among patients with SCD. Most of the studies that assessed the seroprevalence of B19 V included in this meta-analysis were from Africa, which is consistent with the distribution of SCD-prone areas [4]. The present study investigated the seroepidemiological profiles of SCD patients who were tested positive for IgG and\or IgM B19 V antibodies.

Existing evidence from the reviewed studies showed that nearly half of the SCD patients were exposed to the B19 V. Several factors could explain the high prevalence rate of B19 V infection among patients with SCD such as the nature of tropism to red blood precursor cells, easy transmissibility of the virus, lack of preventive vaccination against B19 V, and the increased frequency of blood transfusion among the SCD population [7, 16].

When we examined the heterogeneity among the studies, we found that the seroprevalence of B19 V showed regional epidemiological differences. This finding was also reported by previous reviews done by Broliden et al. and Qiu et al. [8, 58]. In the developing countries, the seroprevalence tends to be a little higher because of the poor and overcrowded living condition [8]. Also, we found that the B19 V seroprevalence is dependent on age—it rises from childhood to the elderly. Effect of age of presentation was supported by previous reviews [8, 11, 59]. They showed that the frequency of B19 V infection increases steadily with age. However, the seroprevalence rise with years shown in Figure 3 did not reach the statistical significance level.

Other sociodemographic characteristics of the patients could explain the heterogeneity found between the included studies [17, 52]. However, a few of the included studies have assessed the association between these sociodemographic characteristics and the risk of B19 V infection. These studies demonstrated that frequent hospitalization, having siblings with acute B19 V infection, and poor socioeconomic conditions such as overcrowding and lack of sanitation are implicated in the burden of B19 V infection [48, 50–52].

The findings of this study need to be considered in the context of some limitations. The inclusion of studies published only in English may compromise representativeness. We could not assess the prevalence of B19 V infection by viral DNA detection because few of the included studies used PCR for B19 V detection. As well, because of the lack of uniformity across studies, we did not assess the possible risk factors of B19 V infection among patients with SCD.

## 5. Conclusions

We have summarized data of several studies exploring the seroprevalence of B19 V among patients with SCD in this systematic review and meta-analysis. B19 V infection occurs with increased frequency in patients with SCD. The results of this study could have an important implication for further controlling transmission and could provide a reference for the management of patients with SCD. Healthcare providers need to be aware of the clinically important association between B19 V infection and SCD to ensure effective management.

## Figures and Tables

**Table 1:** Seroprevalence of B19 V IgG stratiﬁed according to subgroups.

Subgroup		No. of studies	Patients, n/N	Pooled prevalence (%)	*I* ^2^	*t* value; degree of freedom (*P* value)	*X* ^2^ (*P* value)
Continent	Africa	7	569/1008	55.5%	97.04%	−**3.1**; **6** (*P*=0.024P = )	**140.4** (*P*=0.001)
	Americas	6	546/1594	39.0%	96.09%	−**3.2**; **5** (*P*=0.001)	
	Asia	2	160/288	54.9%	97.41%	−**2.2**; **1** (*P*=0.270P = )	

Age group	Children	9	857/2189	42.0%	98.00%	−**4.5**; **8** (*P*=0.002)	**102.1** (*P*=0.001)
	All	5	310/551	56.5%	86.00%	−**5.9**; **5** (*P*=0.004)	
	Adults	1	108/150	72.0%	Na	Na	

## Abbreviations

SCD: Sickle-cell disease
B19 V: Parvovirus B19
ACS: Acute chest syndrome
TAC: Transient aplastic crisis
HKSJ: Hartung–Knapp–Sidik–Jonkman.

## Supplementary Materials

Supplementary Materials Table 1: descriptive summary of the studies included in the review.
