# Supplementary material for: A Meta-Analysis on the Seroprevalence of Parvovirus B19 among Patients with Sickle Cell Disease
Source: Biomed Res Int. 2019 Dec 9;2019:2757450. doi: 10.1155/2019/2757450 (PMC6925911; doi:10.1155/2019/2757450)
Supplement: Supplementary Materials — Table 1: descriptive summary of the studies included in the review. [file 2757450.f1.docx]

**Table 1**: **Descriptive summary of the studies included in the review:**

| **Authors, year** | **Country** | **No. of SCD Patients** | **Age group** | **No. of patients with positive IgG** | **No. of patients with positive IgM** | **Quality assessment (risk of bias)*** |
| --- | --- | --- | --- | --- | --- | --- |
| Tizeba 2017 | Tanzania | 90 | Children | Na | 7 | 2 |
| Alao O.O 2009 | Nigeria | 200 | Children | 79 | Na | 1 |
| Iheanacho 2014 | Nigeria | 150 | All | 92 | 8 | 0 |
| Regaya 2007 | Tunisia | 46 | All | 26 | 4 | 1 |
| Makhlouf, 2015 | Egypt | 100 | Children | 44 | 30 | 1 |
| Ayolabi 2017 | Nigeria | 68 | All | Na | 10 | 1 |
| Iwalokun 2012 | Nigeria | 64 | All | Na | 10 | 0 |
| Girei 2010 | Nigeria | 200 | Children | 79 | 7 | 1 |
| Bamidele 2013 | Nigeria | 73 | All | 45 | 13 | 1 |
| Mamman 2012 | Nigeria | 239 | Children | 204 | Na | 0 |
| Obeid E. 2011 | Saudi Arabia | 138 | All | 52 | 4 | 0 |
| Sharaf 2017 | Bahrain | 150 | Adults | 108 | 6 | 1 |
| Hankins 2016 | USA | 330 | Children | 124 | Na | 0 |
| Smith-Whitley 2004 | USA | 633 | Children | 187 | Na | 0 |
| Zimmerman 2003 | USA | 102 | Children | 54 | Na | 1 |
| Serjeant 1993 | Brazil | 107 | Children | 33 | Na | 0 |
| Slavov 2012 | Brazil | 144 | All | 95 | Na | 1 |
| Furtado 2015 | Brazil | 278 | Children | 53 | 5 | 0 |

***Na*: no available data. * Risk of bias: 0-3: low risk, 4-6 : moderate risk, 7-9: high risk**
